# Probiotics for the Management of Infectious Diseases: Reviewing the State of the Art

**DOI:** 10.3389/fmicb.2022.877142

**Published:** 2022-04-28

**Authors:** Cato Wiegers, Linda H. M. van de Burgwal, Olaf F. A. Larsen

**Affiliations:** Athena Institute, Vrije Universiteit Amsterdam, Amsterdam, Netherlands

**Keywords:** probiotics, infectious diseases, state of the art, patents, clinical trials

## Abstract

This review aims to provide insight into the potential of probiotics as a clinical modality targeted at infectious diseases by creating a comprehensive overview of the state of the art of research and development efforts as shown by patents and clinical trials of the past 20 years. Data were retrieved from patent and clinical trial databases to reflect the long- and short-term developments of probiotics research. The data were analyzed to extract information on the total number of patents and trials for each indication, application date and location, and applicant/sponsor type. A total of 80 infectious diseases were investigated, precipitating in 789 patents and 602 clinical trials for 67 indications studied as targets of probiotics. An increasing trend was seen for the number of patents and clinical trials that were applied for since 1999 with the highest number of patents and clinical trials targeted to digestive tract, respiratory, and urogenital indications. Overall, research demonstrated a substantial interest in probiotics targeting infectious diseases, which was in line with reported unmet needs and global probiotics sales estimates. However, the declining rate of translation from patents to clinical trials indicates that there are some barriers obstructing the research process.

## Introduction

Even as late as 2019, infectious diseases such as lower respiratory tract infections, diarrheal diseases, and tuberculosis were still in the top 10 global causes of death and/or disability-adjusted life years (DALYs; Global Health Observatory, 2021). Previous outbreaks of severe acute respiratory syndrome (SARS), Middle East respiratory syndrome (MERS), and the current COVID-19 pandemic have demonstrated that zoonotic diseases are on the rise (Skowron et al., 2022). Moreover, the incidence and burden of lower respiratory tract infections are predicted to increase in our aging population, which can have detrimental effects on healthcare systems if the situation remains unchanged (Feddema et al., 2021). In 2018, the global financial burden of infectious disease epidemics was estimated to be around US$60 billion annually, and the costs of the ongoing COVID-19 pandemic have been estimated to reach US$16 trillion (Cutler and Summers, 2020; Wellcome, 2021).

Currently, as several years have passed since the start of the pandemic, the world is still suffering the consequences. Despite innovation barriers such as a lack of knowledge about the newly emerging virus (Janse et al., 2021), various vaccines have been developed with sufficient short-term efficacy and effectiveness (Noor, 2021; Whitaker et al., 2022). However, at the moment, the long-term efficacy is still unknown and to date, curative medications have been developed but are not readily available yet (Couzin-Frankel, 2021; Kane and Kounang, 2022). Additionally, according to the WHO, the pandemic has caused disruptions in running immunization programs which may result in a rise of vaccine-preventable diseases in young children (World Health Organization, 2020). Furthermore, a new problem has surfaced in recent decades, which is that antibiotic interventions that have been developed to combat infectious diseases are becoming less effective due to antimicrobial resistance (Peri et al., 2019). Therefore, there is an urgent need for new types of interventional and prophylactic modalities to lower the burden of infectious diseases.

Maintaining and potentially restoring the microbiota of humans is considered a vital aspect of the resilience of humans and animals, protecting against a host of infectious and inflammatory diseases (Larsen and van de Burgwal, 2021). Probiotics, defined as “live microorganisms which when administered in adequate amounts confer a health benefit on the host” (World Health Organization, 2002, p. 8), have demonstrated potential as a clinical modality for several infectious diseases, possibly including COVID-19 (Lei et al., 2017; Infusino et al., 2020). Research suggests that probiotics can be used for infectious diseases because of their beneficial effects on the microbiota of the host, which in turn may result in various other health benefits (Valdez et al., 2014; Reid, 2017; Liu et al., 2018; Infusino et al., 2020). The most important target for probiotics is, to date, the gut microbiota, as it contributes greatly to the development of the immune system and comprises the largest immune organ of the human body (Ubeda and Pamer, 2012). Nevertheless, promising results have also been obtained for, e.g., the lung microbiota in which probiotics exert their effect through the gut-lung axis (Dumas et al., 2018; de Oliveira et al., 2021).

Some of the effects that probiotics can have by altering the human gut microbiota include increased antiviral activity after vaccination (Yeh et al., 2018; Infusino et al., 2020), and prevention and/or treatment of respiratory tract and urogenital infections through inhibition of bacteria adhesion and increasing mucosal barrier functioning (Reid, 2017; Liu et al., 2018; Stavropoulou and Bezirtzoglou, 2020; Lv et al., 2021; Theodosiou et al., 2021). The importance of the gut microbiota is also highlighted in the case of COVID-19, as this virus does not only affect the respiratory tract but has also been associated with a decrease in gut microbiota diversity due to the interactions between the respiratory and digestive tract termed the gut-lung axis (de Oliveira et al., 2021). While research on the relation between COVID-19 and the gut microbiota is still in its infancy, it has been suggested that the use of probiotics may improve the recovery of hospitalized COVID-19 patients compared to patients that do not use probiotics (Zhang L. et al., 2021). However, many clinical trials are still ongoing, making it difficult to fully assess the benefits of probiotics for COVID-19 (Kurian et al., 2021).

Altogether, probiotics may serve as a promising intervention targeting infectious diseases. Besides its potential clinical efficacy, research has also shown that probiotics for oral consumption are safe (van den Nieuwboer et al., 2014, 2015a,b; Larsen et al., 2017). Consequently, probiotics may become interventional modalities with a relatively high benefit to burden ratio. Concerns on the efficacy still remain due to conflicting results between clinical studies (Suez et al., 2019). These conflicting results may be caused by several research barriers. The effects of probiotics can vary not only between strains but also within strains, for example, because of different dosages or characteristics of the user (Suez et al., 2019). Moreover, probiotic species can exert their effects through different mechanisms; lactic acid bacteria, for example, can affect the gut microbiota through fermentation, communication *via* vesicles, as well as the production of antibiotics (Suissa et al., 2022). On top of that, probiotic efficacy has been shown to be impacted by carrier matrices (Flach et al., 2018). Hence, no general statements should be made regarding the efficacy of probiotics due to the aforementioned effects (Flach et al., 2018; Lebeer et al., 2018). Nevertheless, despite these barriers, the field of probiotics is growing very rapidly. Overall, the current state of the art still remains unclear, and in turn, it is unknown what the most beneficial applications of probiotics are regarding not only COVID-19 but other infectious diseases as well.

An effective strategy to understand the state of the art is to investigate what, if any, intellectual property and clinical trials have been applied for. According to Feddema and Claassen (2018), patents can be used as an early indicator of developments in the market as well as new technologies, while clinical trials provide insight into the development of the medical field. This is further clarified by Janse et al. (2020), stating that patents can be used to measure the amount of early-stage research and provide information on long-term developments in the field of research as they offer protection of intellectual property for 20 years. Clinical trials can be used to measure late-stage research because they are an essential step that needs to be completed before a new product can enter the market, providing information on short-term developments through a cross-sectional overview of active clinical trials at a given point in time (Janse et al., 2020). In this line, this review aims to create a broad and complete overview of the state of the art of probiotics and infectious diseases through the analysis of relevant patents and clinical trials of the past 20 years.

## Materials and Methods

To gain insight into innovation efforts using probiotics as a means to prevent, treat, or alleviate symptoms of infectious diseases, both patent and clinical trial databases were studied. As patent applications are executed to protect the intellectual property of new inventions, they are a measure of output of early-stage, applied research (Janse et al., 2020). Clinical trials are a measure of output of late-stage research, as they are executed near the end of the research and development process before market entry (Ramezanpour et al., 2015; Janse et al., 2020). Taken together, they provide an overview of the development of the research field.

### Infectious Disease Selection and Classification

Patent and clinical trial databases were searched for entries combining probiotics and infectious diseases. To operationalize infectious diseases and ensure the inclusion of a wide set of infectious diseases in this study, literature was consulted to create a comprehensive list of infectious diseases (Shetty et al., 2009; Webber, 2019; University of Utah Health, 2021). A table was constructed in consultation with a medical microbiology expert to create a complete and rigorous basis for this study. A total of 80 infectious diseases and infections including their microbiological classifications were categorized into nine different groups based on their most common clinical symptoms (Supplementary Table 1).

According to Nelson (2014), clinical classification is based on a disease’s most common clinical manifestation, which can be a key point in choosing an adequate treatment. Since this research was focused on treatment as well as prevention, immune response enhancement and symptom alleviation, this classification approach was deemed the most useful compared to other conventional classifications such as microbiological or by mode of transmission. However, due to the nature of some infectious diseases, multiple classification options were possible, such as infectious diarrhea, *Escherichia coli*, and *Clostridium difficile* infections. For these infectious diseases, the most common classification in literature was used. Additionally, some diseases including infectious diarrhea and nosocomial infections can have many possible causative pathogens. In order to include as many as possible, no further specifications have been made and the most common causative pathogens (such as norovirus, rotavirus, *E. coli*, and *Salmonella* spp.) have been listed separately. The classification of systemic symptoms was used according to the definition given by the United States National Library of Medicine (NLM) Medical Encyclopedia for infectious diseases “affecting the entire body, rather than a single organ or body part” (NLM, 2021, para. 1).

### Patent and Clinical Trial Data Collection

To identify relevant patents, this study queried the European Patent Office (EPO) Espacenet database, as it has one of the largest patent coverages of over 130 million patents from many different countries and organizations worldwide (Jürgens and Herrero-Solana, 2015). To identify relevant clinical studies, the EU clinical trials register (CTR.eu), the United States NLM database (CT.gov), and the International Clinical Trials Registry Platform (ICTRP) were queried, as these are regularly updated and contain records of clinical trials from 202 countries, covering a total of 17 different clinical trial databases (World Health Organization, 2021). The selection of these databases is in line with previous research in this field (Ramezanpour et al., 2015; Janse et al., 2020; Neevel et al., 2020).

Relevant patent codes were selected with the guidance of experts from the Dutch Patent Office, a part of the Netherlands Enterprise Agency (RVO). To ensure no relevant patents were left out from the search results, both International Patent Classification (IPC) codes and the Cooperative Patent Classification (CPC) codes were used (Table 1). Search queries were formulated for each infectious disease, consisting of the patent classification codes displayed in Table 1 (Espacenet, 2019) followed by the name of the disease and its common name or abbreviation where applicable.

**Table 1:** Overview of included patent classification codes.

CPC/IPC code	Description Espacenet
A61K2035/115	“Probiotics”
A61K35/741	“Probiotics (probiotic yeast, e.g., saccharomyces)”
A61K35/742	“Spore-forming bacteria, e.g., *Bacillus coagulans*, *Bacillus subtilis*, clostridium or *Lactobacillus sporogenes*”
A61K35/744	“Lactic acid bacteria, e.g., enterococci, pediococci, lactococci, streptococci or leuconostocs”
A61K35/745	“Bifidobacteria”
A61K35/747	“Lactobacilli, e.g., *L. acidophilus* or *L. brevis*”
A23L33/135	“Bacteria or derivatives thereof, e.g., probiotics”

A similar approach was taken for the data collection of clinical trials; individual queries were formulated for each infectious disease. To ensure the clinical trials were focused on probiotic treatments, search queries in clinical trial databases also included the following list of the most frequently used probiotic microorganisms: “Probiotic” OR “Probiotics” OR “lactobacillus” OR “streptococcus” OR “bifidobacterium” OR “enterococcus” OR “Escherichia” OR “e coli” OR “saccharomyces” OR “lactic acid bacterium.” For both types of data, search queries were tested extensively to optimize strings. It was found that the use of wildcard operators (*) either did not make a difference in the number of relevant results or caused a higher number of inaccurate results, which is why they were not included. Final database searches were performed on November 15, 2021.

### Patent and Clinical Trial Data Selection

Patents and clinical trials were checked for suitability by manual screening of titles. For patents, a second screening of abstracts, descriptions, and claims was executed in the Espacenet database to ensure inclusion criteria were met. For both patents and clinical trials, the following inclusion criteria were utilized:

- Published from 01.01.1999 until 15.11.2021.
- Containing a description of live probiotics only (no killed microorganisms or derivatives thereof) for oral ingestion.
- Relating to the enhancement of post-vaccination immune response, treatment, prevention, or alleviation of symptoms of infectious diseases.
- For patents: not solely describing a production method or technology.
- For clinical trials: following an interventional study design.

The timescale of 1999 to 2021 was chosen to be able to provide a scope of the past 20 years. Because of the 18 months delay between patent filing and publication (European Patent Office, 2021) as well as COVID-19-related disruptions in research, no definite conclusions could be made from May 2020 onwards. Additionally, patents or clinical trials describing the use of killed microorganisms or derivatives thereof were excluded because these products are not in accordance with the definition of probiotics as stated by the WHO.

### Patent and Clinical Trial Analysis

Data analysis was mainly performed in Microsoft Excel, and for further patent analysis, full patent documents were consulted in the Espacenet database. Focal points in the patent and clinical trial analysis were the number of results that were included, the type of applicant or sponsor (industrial, academic, individual, government, or a collaboration), year, and geographical location. To determine the number of results for each infectious disease, individual patent and clinical trial entries were studied and coded with the names of all indications that were mentioned. For the analysis, indications were categorized by clinical manifestation to increase the clarity of visualizations. Applicant and sponsor types were coded in a similar manner through coding of each included patent and clinical trial, either based on extracted data or further inspection of full patent documents when necessary. Application or start years were readily indicated, and the countries of patent application or clinical trial execution were determined through the country (or organization) codes of each patent, including all patent numbers listed under “also published as,” or the indicated clinical trial locations, respectively.

## Results

### Patent and Clinical Trial Inclusion

**Figure 1:**
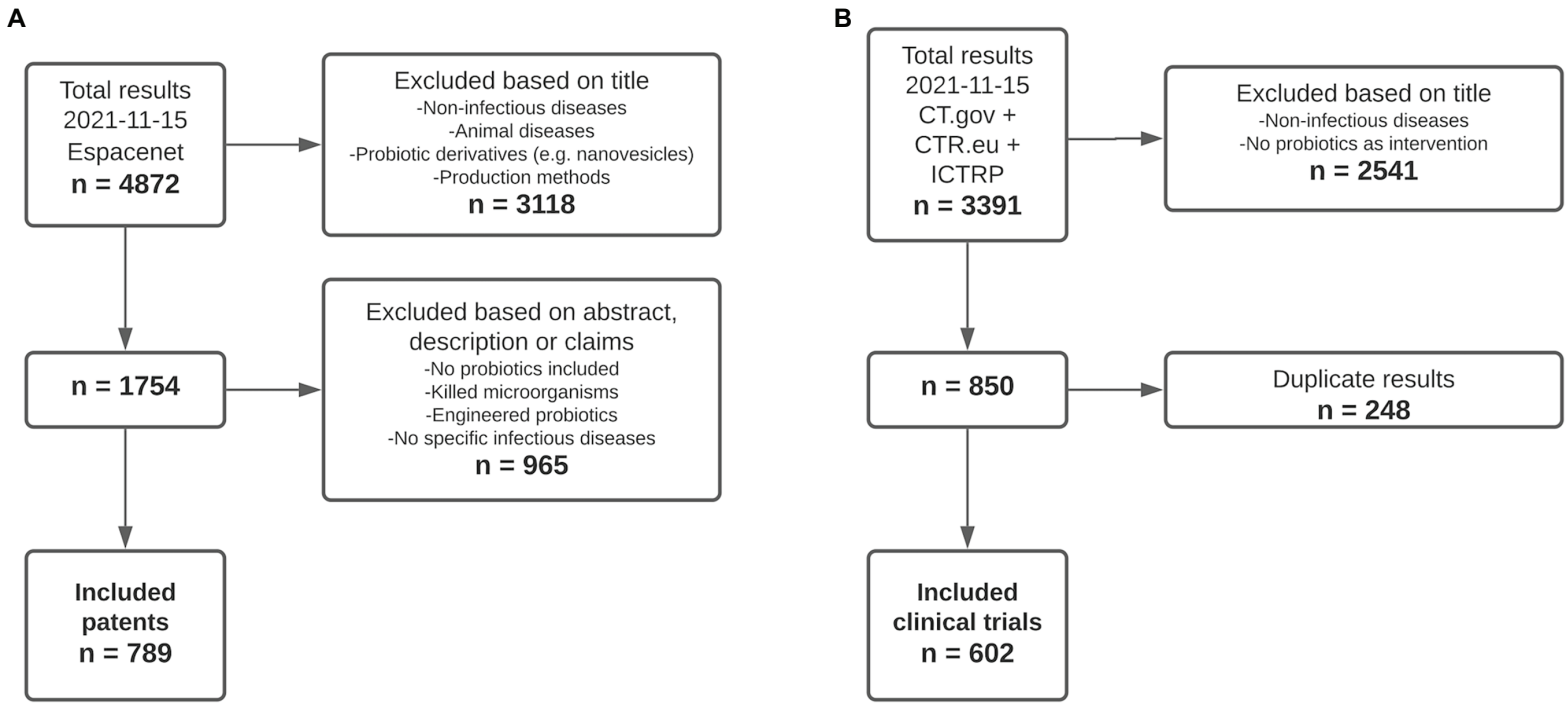
A total of 789 patents and 602 clinical trials were included for analysis. **(A)** Patents. *n* = number of results retrieved from Espacenet. **(B)** Clinical trials. *n* = number of results retrieved from ClinicalTrials.gov (CT.gov), ClinicalTrialsRegister.eu (CTR.eu), and the International Clinical Trials Registry Platform (ICTRP).

Out of a total of 4,872 patent documents retrieved from Espacenet for all relevant infectious diseases, 789 were included for analysis (Figure 1A). The main reasons for exclusion were that patents described production processes, other types of diseases including chronic and autoimmune, the use of probiotic derivatives instead of living organisms, or did not mention probiotics at all. For the included patents, analysis consisted of the indicated infectious disease(s), the country of application, the type of applicant, and the application date. Regarding clinical trials, out of the 3,391 trials that were found studying the relevant infectious diseases, 602 were included for analysis from CT.gov, CTR.eu, and the ICTRP (Figure 1B). Clinical trials were excluded if the indication stated in the title was not an infectious disease or if the specified intervention did not include probiotics. The analysis of included clinical trials consisted of the number of trials focusing on each infectious disease, the location, type of sponsor, and starting year.

### Patent and Clinical Trial Trends

**Figure 2:**
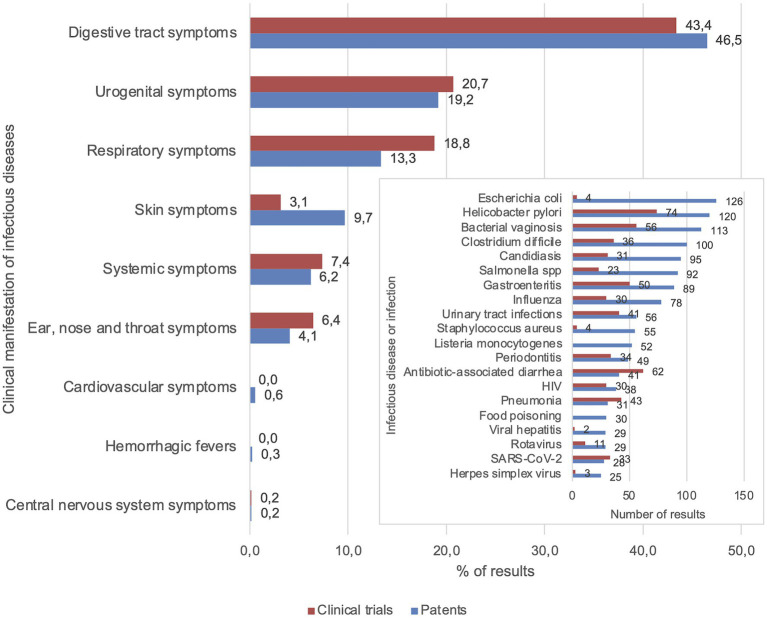
Focal points of patents and clinical trials were digestive tract, urogenital, and respiratory symptoms. Percentages of patent and clinical trial results for each category of infectious diseases based on clinical manifestation. The 20 infectious diseases that were mentioned most frequently in patents are displayed in the insert graph (see Supplementary Figure 1 for complete graph).

The largest numbers of patents were related to infectious diseases with digestive tract (46.5%) and urogenital symptoms (19.2%) including *E. coli* (*n* = 126), *Helicobacter pylori* (*n* = 120), bacterial vaginosis (*n* = 113), and *C. difficile* (*n* = 100), see Figure 2. The 5 infectious diseases with the highest numbers of results were all digestive tract and urogenital infections, whereas the top 10 also included upper respiratory tract (influenza, *n* = 74) and mouth infections (periodontitis, *n* = 49). Contrastingly, few relevant patents were found for most parasitic infections, zoonoses, tropical, and newly emerging infectious diseases (*n* = 35 in total). From the included clinical trials, the majority was focused on infectious diseases with digestive tract (43.4%), urogenital (20.7%), and respiratory symptoms (18.8%), with the highest numbers of results for *H. pylori* infection (*n* = 74), antibiotic-associated diarrhea (*n* = 62), and bacterial vaginosis (*n* = 56; Figure 2). This was followed by pneumonia (*n* = 43) and urinary tract infections (*n* = 41). Finally, 33 clinical trials and 28 patents were focused on probiotic applications for COVID-19.

**Figure 3:**
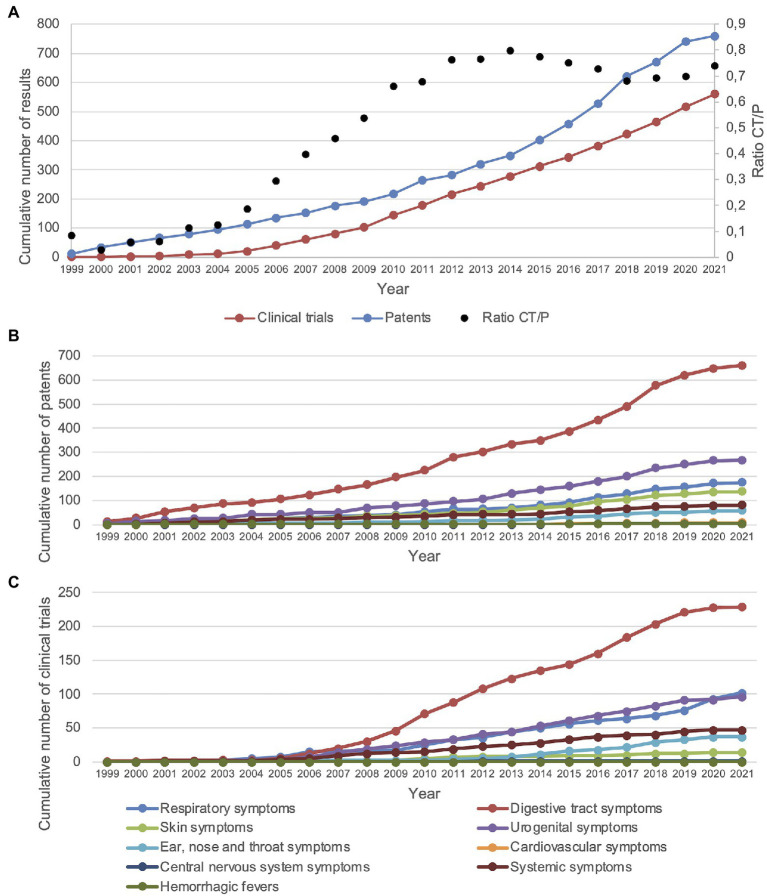
Patents and clinical trials showed an increasing trend since the year 2000. **(A)** CT/P = clinical trials/patents. Displayed are the cumulative number of patents and clinical trials as well as the ratio of clinical trials and patents. **(B)** Cumulative number of patents for each group of clinical symptoms of infectious diseases. **(C)** Cumulative number of clinical trials for each group of clinical symptoms of infectious diseases. Cardiovascular and central nervous system symptoms are obscured due to overlap with hemorrhagic fevers. The years 2020 and 2021 may not be complete due to the 18-month patent application period and COVID-19-related disruptions.

Regarding the first year of application of each patent and clinical trial, the total numbers of patents and clinical trials showed an increasing trend over the years since 1999 (Figure 3A). When looking at the ratio of the number of clinical trials and patents (CT/P) found in each year, a rising trend can be seen from 2000 to ~2014, followed by a slight decline from ~2014 to ~2018 (Figure 3A). Grouping the infectious diseases described in patents by clinical manifestation, probiotic applications related to digestive tract symptoms have been the most popular since 1999, followed by urogenital symptoms (Figure 3B). For clinical trials, a similar pattern could be seen compared to patents relating to infectious diseases with respiratory tract and urogenital symptoms; however, there was a higher focus on clinical trials with probiotics relating to infectious diseases with respiratory symptoms and a lower focus on those with skin symptoms (Figure 3C). For both patents and clinical trials, probiotic applications relating to infectious diseases with central nervous system, ear, nose and throat, systemic, skin, and cardiovascular symptoms, as well as hemorrhagic fevers, showed less cumulative growth (Figures 3B,C).

### Patent and Clinical Trial Applications

**Figure 4:**
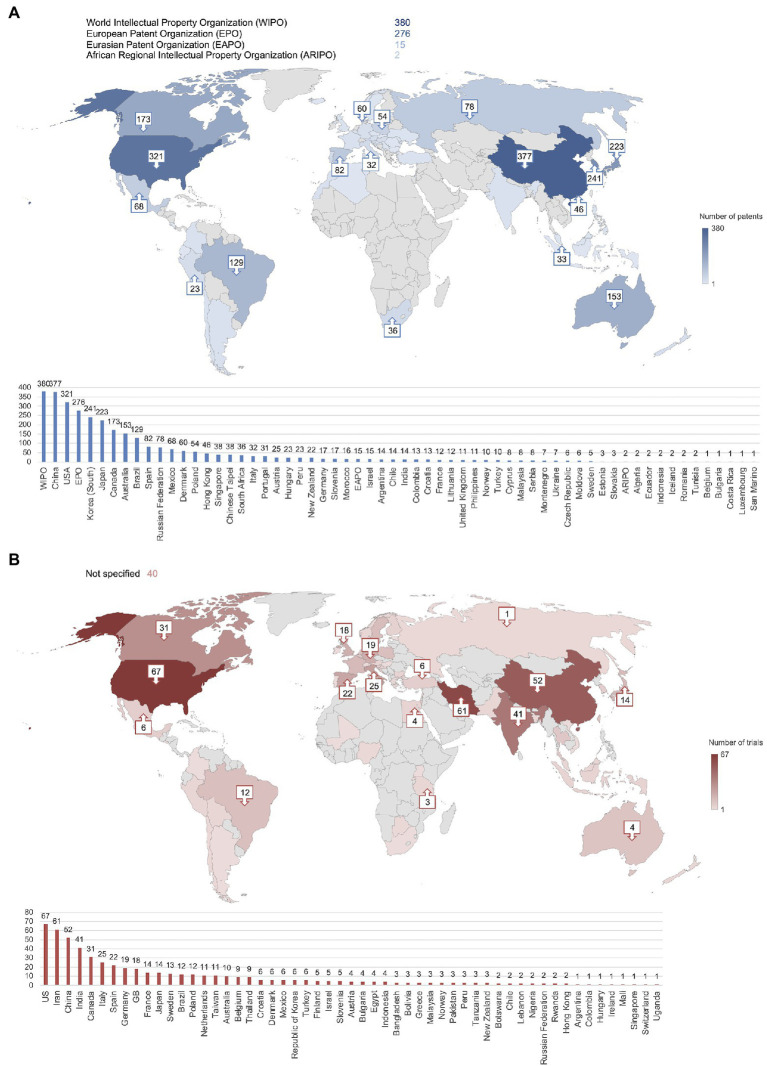
China, the United States, South Korea, and Iran were the most frequently reported locations of patent applications and clinical trials. **(A)** Number of patent applications per country or organization. Total exceeds *n* = 789 due to most patents being applied for in multiple countries. **(B)** Number of clinical trials per country. Numbers not shown on the map are included in the bar graph.

Looking into the application country of each patent, the main locations included Asia and North America with the highest number of patents filed in China (*n* = 377) followed by the United States of America (*n* = 321) and Korea (*n* = 241; Figure 4A). Additionally, 380 patents were applied for at the World Intellectual Property Organization (WIPO), 276 at the EPO, 15 at the Eurasian Patent Organization (EAPO), and 2 at the African Regional Intellectual Property Organization (ARIPO). Figure 4B demonstrates that the main locations of clinical trials were also Asia and North America, with the United States of America as the top location (*n* = 67) followed by Iran (*n* = 61), China (*n* = 52), India (*n* = 41), and Canada (*n* = 31). Overall, clinical trials appeared to be more widespread than patent applications; however, 40 clinical trial records did not specify a location.

**Figure 5:**
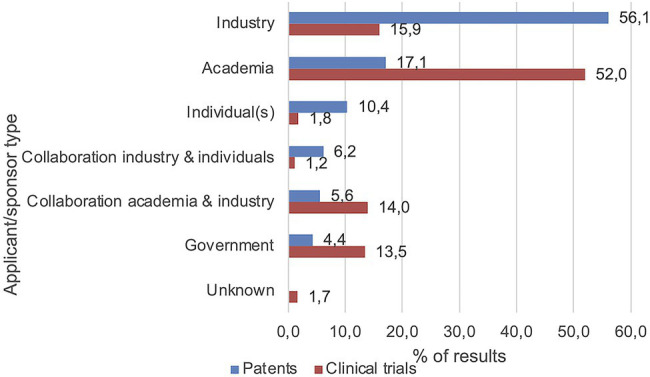
Main applicant/sponsor types included industry and academia. Values represent percentages of the number of included patents and clinical trials, respectively.

Besides the patent application country and trial location, applicant and sponsor types were analyzed (Figure 5). Over half of the patents had industrial applicants (*n* = 437, 56.1%), which was the largest group, followed by academic applicants (*n* = 133, 17.1%), individual applicants (*n* = 81, 10.4%), and collaborations between industry and individuals (*n* = 48, 6.2%) or academia (*n* = 44, 5.6%). The lowest number of patents was applied for by governmental organizations (*n* = 34, 4.4%). Concerning the sponsors/collaborators of the clinical trials, over half were funded by academic parties (*n* = 313, 52.0%). The second-largest sponsor group consisted of industrial parties (*n* = 96, 15.9%) followed by collaborations between academia and industry (*n* = 84, 14.0%) and governmental parties (*n* = 81, 13.5%). The lowest number of clinical trials was sponsored by individuals (*n* = 11, 1.8%) and collaborations between industry and individuals (*n* = 7, 1.2%). Some trial records did not specify a sponsor or collaborator (*n* = 10, 1.7%).

## Discussion

This study presents an overview of the state of the art regarding patents and clinical trials for probiotics targeted toward infectious diseases. The research landscape consisted of 789 patents and 602 clinical trials mainly targeted toward infectious diseases with digestive tract, urogenital, and respiratory symptoms. The rapid increase in cumulative patents and clinical trials that was observed from the year 1999 to the year 2021 strongly suggests that probiotics as clinical modalities for infectious diseases have gained considerable interest, mainly in Asia and North America, from industrial and academic parties.

It was found that over the past 20 years, the cumulative increase in the number of patents describing probiotic applications for infectious diseases differed slightly from the increase in the number of clinical trials. The cumulative number of patents seems to reach the beginning of a plateau, whereas the cumulative number of clinical trials seems to be increasing up to the year 2021 without reaching a plateau. According to Fernald et al. (2013), the cumulative number of patents over time can be compared to the so-called technology saturation curve (S-curve) originally described by Ernst (1997). This S-curve demonstrates technological change within an industry in four stages: Emerging, Growth, Maturity, and Saturation (Weenen et al., 2013). Based on the data we collected, it may be suggested that the probiotics industry as reflected by the cumulative number of patents has currently entered the maturity stage. However, this has yet to be confirmed by data from future years and may be subject to change due to delays caused by the COVID-19 pandemic and the 18-month patent application process.

Regarding clinical trials, the cumulative number over the past 20 years showed a less pronounced growth without seeming to reach a plateau, suggesting that based on the S-curve, the maturity stage has not yet been reached. This “lag time” between patents and clinical trials may have multiple causes. Firstly, van de Burgwal et al. (2018) state that patent application is normally an earlier step in the research process than clinical trial execution. Secondly, the patent application process is relatively less difficult and expensive than the execution of a clinical trial; new probiotic products may be abandoned during early-stage development due to strict regulations on probiotics research (van den Nieuwboer et al., 2016; Binda et al., 2020). This in turn could cause a gap between the number of patents that are applied for and the number of clinical trials that are executed.

Infectious diseases with digestive tract symptoms showed the highest growth for patents as well as clinical trials, making these the most popular indications overall. The high focus on digestive tract symptoms is not surprising, as the digestive tract is the place where probiotics can directly interact with the gut microbiota of the host as well as pathogens (Cazorla et al., 2018; Lv et al., 2021; Taverniti et al., 2021). Moreover, these findings are in agreement with a previous study by van den Nieuwboer et al. (2016). In this study, unmet needs and research priorities according to key opinion leaders (KOLs) in the field of probiotics showed that for people of all ages, conditions relating to the digestive tract such as antibiotic-associated diarrhea had a high priority (van den Nieuwboer et al., 2016).

The second and third highest numbers of patents and clinical trials were found for infectious diseases with urogenital and respiratory symptoms, respectively. Susceptibility to these types of diseases has been linked to the microbiome composition of young children and older adults (Horwitz et al., 2015; Unger and Bogaert, 2017). The study on unmet needs and research priorities according to KOLs (van den Nieuwboer et al., 2016) showed that there was a medium research priority for respiratory infections in infants and children, and a low research priority in adults. Overall, this medium to low prioritization appears to be in line with the percentages of patents and clinical trials that were found. The only exception is the high number of clinical trials studying the effects of probiotics on COVID-19 (*n* = 33) that have been executed in the last 2 years, which is not surprising considering that the COVID-19 pandemic only started after publication of the research by van den Nieuwboer et al. (2016). Regarding the research priority of infectious diseases with urogenital symptoms, it is difficult to compare the findings of this study with those of van den Nieuwboer et al. (2016) because these are not specifically articulated, either due to low prioritization or the use of alternative terminations.

Use of termination and specification was further investigated, showing that over half of the data entries included in this review did not specify the strain of the probiotic that was used. Data were sampled by randomly selecting 2–5 records of patents and clinical trials, respectively, for each of the top 24 most frequently studied infectious diseases. The number of patents and clinical trials that were sampled for each infectious disease depended on the number of results included in the final dataset to increase the accuracy of the sample, totaling approximately 10% of the data. Out of the 80 sampled patents, ~49% (*n* = 39) specified one or multiple probiotic strains and out of 62 patents, ~39% (*n* = 24) specified a probiotic strain or product with traceable composition. The lack of specification can be considered a barrier in the research process, as the effects of a probiotic can differ vastly between strains (Hill et al., 2014; Suissa et al., 2022), making it unclear which probiotic strain or strains are responsible for the reported health effects disclosed in patents and clinical trials, and negatively impacting the replicability of probiotic research.

The clinical trials included in this review were mainly sponsored/executed by academic institutions, whereas patents were mainly applied for by industrial parties. The contributions of the three main applicant/sponsor groups (industry, academia, and government) were compared for each indication type following methods described in previous research (van de Burgwal et al., 2016). For all nine categories of infectious diseases, similar divisions were found between the three groups of patent applicants and clinical trials sponsors. This is in accordance with the notion that clinical trials provide insight into research trends with academic parties being more focused on fundamental research, whereas patents can be considered a reflection of the market with industrial parties that are more focused on applied research and commercialization (Feddema and Claassen, 2018; Janse et al., 2020).

The International Probiotics Association (IPA) reported that in 2019, China ranked highest in sales of probiotic supplements and yoghurts, followed by the United States and Europe (International Probiotics Association, 2019). Analogous to these sales estimates, it was found that Asia and North America were the most popular locations for clinical trials and patent applications. According to Neevel et al. (2020), the location of patent applications is closely related to expected profit, as the costs of patent filing and maintenance are very high. Lower-income countries were less frequently reported as the patent application and clinical trial locations. Reid et al. (2018) have stated that many people in these countries do not have access to probiotic products. However, the IPA highlights that these particular people may benefit the most from probiotics as low hygiene and lack of clean water cause a surge in infectious disease prevalence (International Probiotics Association, 2015).

When interpreting the findings of this study, several limitations need to be considered. Firstly, due to the patenting process taking up to 18 months from application to publication (European Patent Office, 2021), no definite conclusions can be made about the most recent data. In a similar light, it seems most likely that the COVID-19 pandemic has affected the data that was collected in this study. However, as the pandemic is still ongoing its true effects cannot be determined yet. To ensure high-quality results, our research methodology was validated by research experts in the fields of patents, clinical trials, infectious diseases, and (medical) microbiology. To allow for a thorough investigation of the state of the art, the choice was made to focus on a broad scope of indications for probiotics only, leaving pre- and postbiotics out of the picture.

For further research, it may be beneficial to investigate details such as the strain and dosage of probiotics that were indicated in the relevant patents and clinical trials, as well as the mechanisms of action of the respective probiotics. Additionally, alternative interventions that target the gut microbiota should not be disregarded, like fecal microbiota transplants (FMT), which can be considered probiotics “in extremo” and can be, among others, helpful to restore dysbiosis caused by *C. difficile* infections (Ser et al., 2021). Interestingly, the effects of FMT after antibiotics are impaired by the use of a multistrain probiotic (Suez et al., 2018), which demonstrates again the need for more research into strain specific effects (Hill et al., 2014).

To conclude, the increasing amount of research as reflected by the numbers of patents and clinical trials of the past 20 years suggests the acknowledgement of the potential of probiotics as applications for the management of infectious diseases. The current state of the art coincides with reported research priorities, expected sales and market growth (van den Nieuwboer et al., 2016; International Probiotics Association, 2019). However, evidence suggests that research and development at the intersection of probiotic products and infectious diseases are slowing down. This may be due to the natural progression of the technology life cycle or external factors such as regulations on probiotics research. Either way, in light of the COVID-19 pandemic and any future infectious disease outbreaks caused by our changing society (Hersh et al., 2012; Zhang D. et al., 2021; Skowron et al., 2022), it is important to stimulate the progression of the state of the art of clinical modalities targeting infectious diseases. As highlighted before (Larsen and van de Burgwal, 2021), such progression should consider the effect of changes to microbial communities across ecosystems.

## Author Contributions

CW: analysis and writing with guidance of LB and OL. LB: reviewing and editing. OL: conceptualization and reviewing and editing. All authors contributed to the article and approved the submitted version.

## Funding

The contribution of LB is financed by the project Preparedness for Emerging Infectious Diseases [with project number VI.Veni.201S.044 of the research program Veni SGW which is financed by the Dutch Research Council (NWO)]. The contribution of CW is financed by Yakult Nederland B.V.

## Conflict of Interest

LB is a consultant for several commercial parties in the field of probiotics and life sciences; none of her advising practices are related to or in conflict with the content of this research. OL is Senior Manager Science at Yakult Nederland B.V.

The remaining author declares that the research was conducted in the absence of any commercial or financial relationships that could be construed as a potential conflict of interest.

## Publisher’s Note

All claims expressed in this article are solely those of the authors and do not necessarily represent those of their affiliated organizations, or those of the publisher, the editors and the reviewers. Any product that may be evaluated in this article, or claim that may be made by its manufacturer, is not guaranteed or endorsed by the publisher.
