# Supplementary material for: Probiotics for the Management of Infectious Diseases: Reviewing the State of the Art
Source: Front Microbiol. 2022 Apr 28;13:877142. doi: 10.3389/fmicb.2022.877142 (PMC9096241; doi:10.3389/fmicb.2022.877142)
Supplement: Supplementary file 1 [file Table_1.DOCX]

Supplementary Material

# Overview of all included infectious diseases and infections

**Supplementary Table 1. Included infectious diseases.**

| **Clinical manifestation** | **Infectious disease or infection** | **Microbiological classification** | **Including** |
| --- | --- | --- | --- |
| **Respiratory symptoms** | Bronchitis | Bacteria, viruses |  |
|  | Pneumonia | Bacteria, viruses, fungi |  |
|  | *Bordetella pertussis* | Bacteria |  |
|  | *Corynebacterium diphtheriae* | Bacteria |  |
|  | *Mycobacterium tuberculosis* | Bacteria |  |
|  | Adenovirus | Viruses |  |
|  | Influenza | Viruses |  |
|  | MERS^a^ | Viruses |  |
|  | Rhinovirus | Viruses |  |
|  | SARS-CoV-1^b^ | Viruses |  |
|  | SARS-CoV-2^c^ | Viruses |  |
|  |  |  |  |
| **Digestive tract symptoms** | Antibiotic-associated diarrhea | Bacteria |  |
|  | Food poisoning | Bacteria, viruses, parasites |  |
|  | Gastroenteritis | Bacteria, viruses, parasites | Including infectious and travelers’ diarrhea |
|  | Mucositis | Bacteria, viruses, parasites |  |
|  | Peritonitis | Bacteria, fungi |  |
|  | Hookworm | Parasites |  |
|  | *Campylobacter* spp | Bacteria |  |
|  | *Clostridium botulinum* | Bacteria |  |
|  | *Clostridium difficile* | Bacteria |  |
|  | *Escherichia coli* | Bacteria |  |
|  | *Helicobacter pylori* | Bacteria |  |
|  | *Listeria monocytogenes* | Bacteria |  |
|  | *Salmonella* spp | Bacteria |  |
|  | *Vibrio Cholerae* | Bacteria |  |
|  | Norovirus | Viruses |  |
|  | Rotavirus | Viruses |  |
|  | *Giardia duodenalis* | Parasites |  |
|  | *Schistosoma* spp | Parasites |  |
|  | *Taenia* spp | Parasites |  |
|  |  |  |  |
| **Skin symptoms** | Mycosis | Fungi |  |
|  | *Bacillus anthracis* | Bacteria |  |
|  | MRSA^d^ | Bacteria |  |
|  | *Mycobacterium leprae* | Bacteria |  |
|  | *Staphylococcus aureus* | Bacteria |  |
|  | *Streptococcus* spp | Bacteria |  |
|  | *Yersinia pestis* | Bacteria |  |
|  | Herpes simplex virus | Viruses |  |
|  | Measles virus | Viruses |  |
|  | Monkeypox virus | Viruses |  |
|  | Mumps virus | Viruses |  |
|  | Rubella virus | Viruses |  |
|  | Varicella-zoster virus | Viruses |  |
|  | *Leishmania* spp | Parasites |  |
|  |  |  |  |
| **Urogenital symptoms** | Bacterial vaginosis | Bacteria |  |
|  | Candidiasis | Fungi |  |
|  | Urinary tract infections | Bacteria |  |
|  | *Chlamydia trachomatis* | Bacteria |  |
|  | *Neisseria gonorrhoeae* | Bacteria |  |
|  | *Treponema pallidum* | Bacteria |  |
|  | HPV^e^ | Viruses |  |
|  |  |  |  |
| **Ear, nose and throat symptoms** | Otitis | Bacteria, viruses |  |
|  | Periodontitis | Bacteria |  |
|  |  |  |  |
| **Cardiovascular symptoms** | Endocarditis | Bacteria, fungi |  |
|  | Myocarditis | Bacteria, viruses, fungi, parasites |  |
|  | Pericarditis | Viruses |  |
|  |  |  |  |
| **Central nervous system symptoms** | Encephalitis | Viruses |  |
|  | Meningitis | Bacteria, viruses, fungi |  |
|  | Rabies lyssavirus | Viruses |  |
|  |  |  |  |
| **Systemic symptoms** | Nosocomial infections | Bacteria, fungi |  |
|  | Typhus | Bacteria |  |
|  | *Borrelia* spp | Bacteria |  |
|  | *Brucella* spp | Bacteria |  |
|  | *Clostridium tetani* | Bacteria |  |
|  | *Coxiella burnetii* | Bacteria |  |
|  | Chikungunya virus | Viruses |  |
|  | Epstein-Barr virus | Viruses |  |
|  | Hantavirus | Viruses |  |
|  | Henipavirus | Viruses | Including Nipah and Hendra viruses |
|  | HIV^f^ | Viruses |  |
|  | Viral hepatitis | Viruses |  |
|  | West Nile virus | Viruses |  |
|  | Yellow fever virus | Viruses |  |
|  | Zika virus | Viruses |  |
|  | *Plasmodium* spp | Parasites |  |
|  | *Toxoplasma gondii* | Parasites |  |
|  | *Trypanosoma cruzi* | Parasites |  |
|  |  |  |  |
| **Hemorrhagic fevers** | Dengue virus | Viruses |  |
|  | Ebolavirus | Viruses |  |
|  | Lassa virus | Viruses |  |

^a^Middle Eastern Respiratory Syndrome
^b^Severe Acute Respiratory Syndrome Coronavirus 1
^c^ Severe Acute Respiratory Syndrome Coronavirus 2
^d^Methicillin-resistant *Staphylococcus aureus*^e^Human Papilloma Virus
^f^Human Immunodeficiency Virus

#
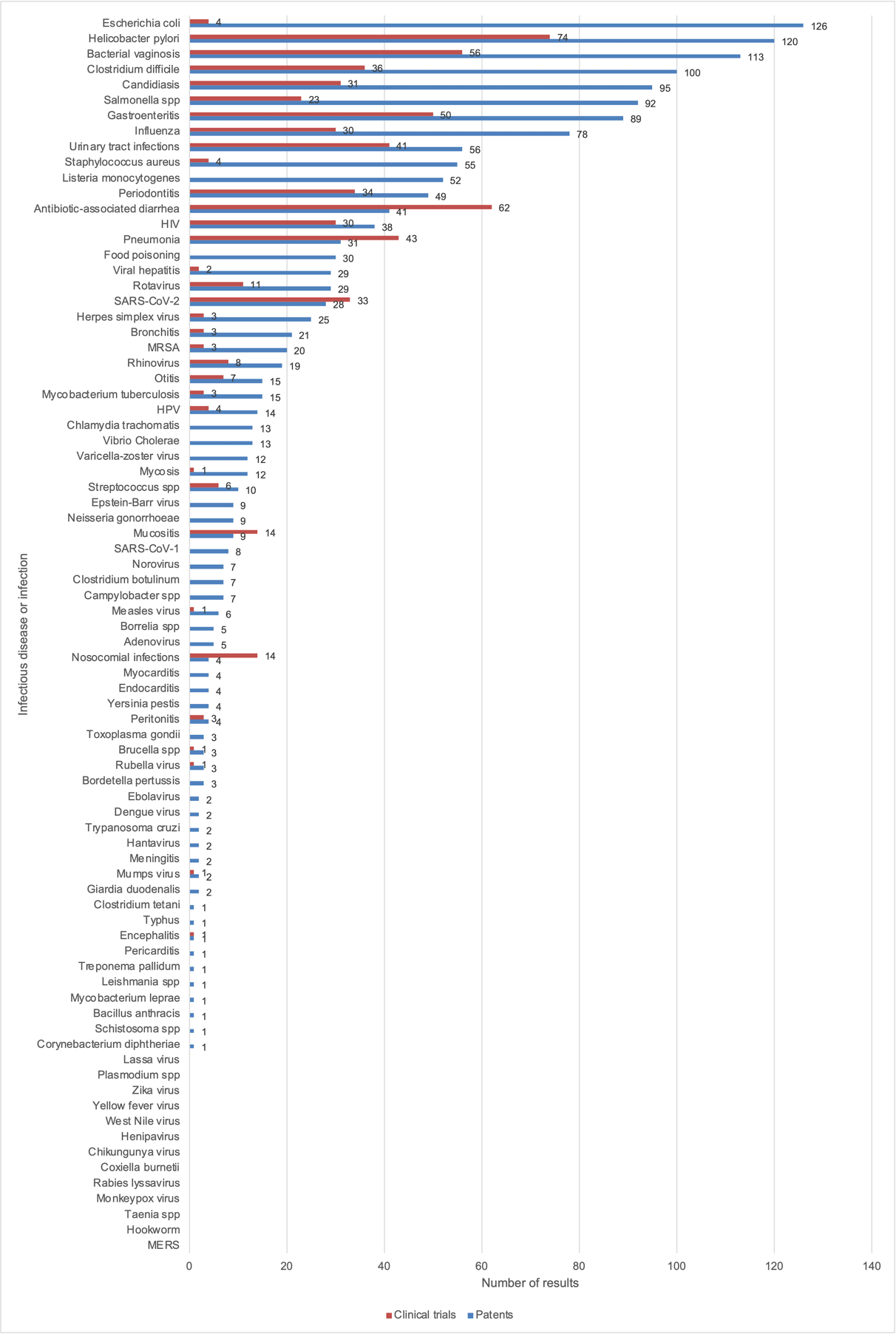
Overview of the numbers of patents and clinical trials focused on each infectious disease

**Supplementary Figure 1. The main focus of patents and clinical trials were *Escherichia coli* and *Helicobacter pylori* infections**. n exceeds the total number of included results due to some patents and clinical trials focusing on multiple indications.
